# Energy demanding RNA and protein metabolism drive dysfunctionality of HIV-specific T cell changes during chronic HIV infection

**DOI:** 10.1371/journal.pone.0298472

**Published:** 2024-10-02

**Authors:** Lisa van Pul, Melissa Stunnenberg, Stefanie Kroeze, Karel A. van Dort, Brigitte D. M. Boeser-Nunnink, Agnes M. Harskamp, Teunis B. H. Geijtenbeek, Neeltje A. Kootstra

**Affiliations:** 1 Amsterdam UMC location University of Amsterdam, Laboratory for Viral Immune Pathogenesis, Amsterdam, The Netherlands; 2 Amsterdam UMC location University of Amsterdam, Department of Experimental Immunology, Amsterdam, The Netherlands; 3 Amsterdam Institute for Infection and Immunity, Amsterdam, The Netherlands; The University of Texas Rio Grande Valley, UNITED STATES OF AMERICA

## Abstract

Antiretroviral treatment of HIV infected individuals cannot eliminate the HIV reservoir and immune control of HIV is rarely seen upon treatment interruption. In long-term non-progressors (LTNP), an effective CD8 T cell response is thought to contribute to be immune control of HIV. Here we studied the transcriptional profile of virus specific CD8 T cells during the asymptomatic phase of disease, to gain molecular insights in CD8 T cell functionality in HIV progressors and different groups of LTNP: HLA-B*57 LTNP, non-HLA-B*57 LTNP and individuals carrying the MAVS minor genotype (rs7262903/rs7269320). Principal component analysis revealed distinct overall transcriptional profiles between the groups. The transcription profile of HIV-specific CD8 T cells of LTNP groups was associated with increased cytokine/IL-12 signaling and protein/RNA metabolism pathways, indicating an increased CD8 T cell functionality. Although the transcription profile of CMV-specific CD8 T cells differed from that of HIV-specific CD8 T cells, with mainly an upregulation of gene expression in progressors, similar affected pathways were identified. Moreover, CMV-specific CD8 T cells from progressors showed increased expression of genes related to effector functions and suggests recent antigen exposure. Our data shows that changes in cytokine signaling and the energy demanding RNA and protein metabolism are related to CD8 T cell dysfunction, which may indicate that mitochondrial dysfunction is an important driver of T cell dysfunctionality during chronic HIV infection. Indeed, improvement of mitochondrial function by IL-12 and mitoTempo treatment, enhanced in vitro IFNγ release by PBMC from PWH upon HIV gag and CMV pp65 peptide stimulation. Our study provides new insights into the molecular pathways associated with CD8 T cell mediated immune control of chronic HIV infection which is important for the design of novel treatment strategies to restore or improve the HIV-specific immune response.

## Introduction

HIV replication and disease progression can effectively be prevented by antiretroviral therapy (ART), however HIV is not eliminated by ART, making life-long treatment necessary. Although the large majority of HIV infected individuals will need ART to control viral replication, studies involving long-term non-progressors (LTNP) show that natural immunological control of HIV replication is possible [1–3]. Moreover, immunological control of HIV has been observed in rare individuals upon treatment interruption [4–7].

CD8 T cells are thought to be an essential component in immunological control of HIV. In acutely infected individuals the decline in viremia coincides with the emergence of HIV-specific CD8 T cells [8, 9]. Furthermore, after depletion of CD8 T cells in SIV-infected macaques there is an increase in viral load and a more rapid disease progression [10, 11]. Moreover, the frequent occurrence of escape mutations in CD8 T cell epitopes also provides evidence that these cells play an important role in control of HIV infection [12–22]. Indeed, HIV-specific CD8 T cells can be detected in most HIV-infected individuals [8, 9, 23], but their ability to control HIV is lost due to functional impairment and exhaustion by chronic antigen exposure, upregulation of inhibitory molecules like PD-1 and Tim-3 and low CD4 T cell counts, in combination with viral escape [12, 15, 18, 24–27].

Recently, T cell dysfunction and exhaustion has been linked to metabolic changes [28–30]. While quiescent CD8 T cells rely mostly on fatty acid dependent oxidative phosphorylation for their survival, T cell receptor triggering of CD8 T cells leads to rapid metabolic reprogramming to glycolysis and increased glucose uptake through upregulation of glucose transporters [31, 32]. However, in chronic infections signaling of inhibitory receptors like PD-1 and CTLA4, inhibit glycolysis which results in a metabolic switch to fatty acid dependent oxidative phosphorylation, which will not suffice in energy demands resulting in poor CD8 T cell functionality [33, 34]. In addition, mitochondrial dysfunction has been observed in exhausted T cells which prevents efficient oxidative phosphorylation [35, 36].

To elucidate mechanisms behind the loss of an effective CD8 T cell response in chronic HIV infection, the gene expression profile of HIV-specific CD8 T cells from individuals with progressive disease or individuals with HLA-B57 as well as non-HLA-B57 individuals who control HIV infection, were analyzed. To determine whether chronic HIV infection is related to an overall disturbed immune function the gene expression profile of cytomegalovirus (CMV)-specific CD8 T cells was determined in the same individuals and compared to CMV-positive healthy controls. Our results indicate that the HIV-specific CD8 T cells from LTNP and progressors have a distinct gene expression profile. The differential gene expression profile of LTNP was associated with increased functionality and included genes related to RNA and protein metabolism pathways. Although CMV specific CD8 T cells showed a distinct gene expression profile consisting of genes mainly upregulated in progressors, the differentially expressed genes were related to the same pathways. In addition, CMV-specific CD8 T cells from progressors showed a gene expression profile related to increased effector functions. The identification of gene profiles related to cytokine/IL-12 signaling and the energy demanding RNA and protein metabolism may be indicative of mitochondrial dysfunctionality as an important driver of T cell dysfunctionality during chronic HIV infection. Indeed, improvement of mitochondrial function by IL-12 and mitoTempo, enhanced in vitro IFNγ release by PBMC from PWH upon HIV gag and CMV pp65 peptide stimulation. Our study provides insights into the mechanisms involved in the natural immune control of HIV and CMV infection in progressors and LTNP, and is important for novel treatment strategies to restore or improve the functionality of the HIV-specific immune response.

## Materials and methods

### Study participants

The Amsterdam Cohort Studies (ACS) on HIV infection and AIDS is a prospective study among men who have sex with men and injecting drug users that started in 1984 [37, 38]. Twenty-six HIV infected participants who were either Human Leukocyte Antigen (HLA) class I type A*02, B*07 or B*57, were selected for this study and these participants were divided into 4 groups: progressors, HLA B*57 LTNP, non-HLA B*57 LTNP and individuals carrying the MAVS minor genotype (MAVS-/-). Individuals carrying other protective HLA class I alleles were excluded. Progressors were diagnosed with AIDS between 6–10 years after seroconversion (SC), and a PBMC sample for analysis was taken at least 4 years after SC and at least 6 months before AIDS diagnosis. HLA B*57 LTNP and non-HLA B*57 LTNP had an AIDS free follow up of more than 10 years and a PBMC sample for analysis was selected at least 6 years after SC. MAVS-/- individuals were homozygous for the minor genotype of rs7262903 and rs7269320 in the MAVS gene and were previously identified in a genome wide association study [39]. A PBMC sample at least 3,5 years after seroconversion when CD4 T cell counts were >200 cells/ul was selected. None of the HIV-infected study participants received effective antiviral therapy before or at the time point of analysis. A group of six CMV seropositive HLA-B*07 carrying healthy blood donors (BD) from the Dutch national blood bank in Amsterdam, the Netherlands (www.sanquin.nl) were included.

The ACS has been conducted in accordance with the ethical principles set out in the declaration of Helsinki. Authors had no access to information that could identify individual participants during or after data collection.

The study was approved by the institutional review board of the Academic Medical Center. Written informed consent was obtained from all participants (MEC 07–182).

### Cellular HIV load

DNA was isolated from MACS isolated CD4 cells using the AllPrep RNA/DNA kit (QIAGEN, Venlo, The Netherlands) and DNA concentrations were determined by Nanodrop. The cell-associated HIV load was determined by qPCR on the LightCycler 480 using the GoTaq qPCR Master Mix (Promega, Madison, WI, USA). Primer sets used were HIV-pol-B (Fw) 5′-TAACCTGCCACCTGTAGTAGCAAAAGAAAT-3′ and Pol-E (Rev) 5′-ATGTGTACAATCTAGTTGCCA-3′. Reactions were carried out using 25ng DNA and 20 μM of each primer. Amplification conditions were as follows a pre-incubation stage at 95°C for 3 min, a pre-amplification stage consisting of two steps, 15 s at 95°C and 15 s at 49°C (2 cycles), amplification stage consisting of 3 steps 10 s at 95°C, 20 s at 58°C, 30 s at 72°C (40 cycles), a melting curve stage consisting of 5 s at 95°C and 1 min at 55°C, and finally a cooling stage of 10 s at 40°C. The 8E5/LAV cell line was used as standard curve in order to quantify the HIV DNA copy number.

A single genome amplification (SGA) was performed when the cellular viral load was below the detection limit of the qPCR. In short, a minimum of 10 PCR reactions were performed using GoTaq polymerase containing 2.5-250ng DNA. Primer sets used were as follows: for primary PCR Pol-F 5′- TTAGTCAGTGCTGGAATCAGG-3′ and Pol-D 5′-GCTACATGAACTGCTACCAGG-3′ were used and for nested PCR; Pol-B and Pol-E. The PCR cycles for both primary and nested PCR reactions were as follows; denaturation 5 min at 94°C followed by 35 cycles of 15 s at 94°C, 30 s at 50°C, 45 s at 72°C and finally followed by 1 cycle of 5 min at 72°C. PCR reactions were visualized on 1% agarose gel. When at least two thirds of reactions were positive, it was presumed that each reaction contained no more than one proviral DNA copy. The proviral HIV-DNA copy number per cell was calculated assuming a concentration of 6pg DNA per cell.

### HLA typing

HLA class I genotyping of all included ACS participants was performed previously by sequence specific primers (SSP) PCR as described elsewhere [40]. BD carrying the HLA-B*07 allele were identified using PCR as described [41]. BD were negative for the HLA-B*57 allele as determined by PCR detecting the rs2395029 in the HCP5 gene region [42].

### Cell sorting of antigen-specific CD8 T cells

Cryopreserved PBMC were thawed and the CD4 T cells were depleted using CD4 MACS Microbeads (Miltenyi Biotec, Bergisch Gladbach, Germany). Virus-specific CD8 T cells in the CD4 T cell depleted cell fraction were stained for 10 minutes at room temperature using PE-labeled MHC class I dextramer carrying HLA-A*02, B*07 or B*57-molecules loaded with immunodominant HIV-gag epitopes SLYNTVATL, GPGHKARVL or KAFSPEVIPMF respectively and APC-labeled MHC class I dextramers carrying HLA-A*02 or HLA-B*07 molecules carrying CMV pp65 epitopes NVLPMVATV and TPRVTGGGAM respectively (Immudex, Virum, Denmark). Next, samples were stained for 20 minutes at 4°C with CD3-Brilliant Violet 510 (BioLegend, San Diego, CA, USA) and CD8- Pacific Blue (BioLegend, San Diego, CA, USA). Virus specific CD8 T cells were sorted on the Becton Dickinson Influx Cell Sorter (Gating strategy is displayed in S6 Fig in S1 File).

### RNA sequencing of antigen-specific CD8 T cells

Sorted virus-specific CD8 T cells were processed using the QIAseq UPX 3’ transcriptome kit (QIAGEN, Venlo, The Netherlands) to generate libraries followed by RNA sequencing. Approximately 300 cells were used for each participant (S1 Table in S1 Data). In brief, cells were lysed and reverse transcribed using oligo-dT primers containing a random unique molecular index (UMI) and a fixed well ID. The cDNA was amplified using PCR and during the PCR indices were added. After PCR the samples were purified. Library preparation QC was performed using TapeStation 4200 (Agilent Technologies, Santa Clara, CA, USA) or Agilent® Bioanalyzer. Based on quality of the inserts and the concentration measurements the libraries were pooled in equimolar ratios. The library pools were quantified using qPCR. The library pools were then sequenced on a NextSeq500 sequencing instrument according to the manufacturer instructions. Raw data was de-multiplexed and FASTQ files for each sample were generated using the bcl2fastq software (Illumina Inc., San Diego, CA, USA). FASTQ data were checked using the FASTQC tool. Trimming of the reads, and UMI grouping was carried out using CLC Genomics Workbench (version 12.0.4) and CLC Genomics Server (version 11.0.3). Reads were mapped to the human reference genome hg38 and annotated using the NCBI RefSeq GRCh38.p11. Reads were filtered for quality and libraries <40000 reads were not included in analysis. Next, libraries were normalized using trimmed mean of M-values (TMM) normalization in RStudio (version 3.6.1). The RNA transcription profile of virus-specific CD8 T cells was generated between August 14 and December 18, 2019.

The dataset generated in this study has been deposited at https://www.ncbi.nlm.nih.gov/geo/ with the accession number GSE197333.

### Differential gene expression analysis and pathway analysis

Differential gene expression analysis was performed using the EdgeR package [43] in RStudio. Genes with a logFC of >1.5 and p-value <0.05 were considered DEGs and were included in pathway enrichment analysis and network analysis. Pathway enrichment analysis of DEGs was performed using the Reactome Pathway browser (version 3.7 release 80) [44] which uses hypergeometric distribution to determine whether certain Reactome pathways are over-represented in the provided list of DEGs. Pathways with an entities p <0.05 were considered significant. Network analysis plots were generated using STRING-db [45], excluding DEG nodes that did not connect to other nodes. Settings were as follows: medium confidence (0.400), MCL clustering with inflation parameter set to 3. Associations between logCPM gene expression values and log_10_ viral load were examined with multivariable linear regression models using the Bioconducter (v.3.18) limma package in Rstudio (v.4.3.3) [46]. Co-variables included in the models were age and CD4+ T-cell count; group (progressors, HLA B*57 LTNP, non-HLA B*57 LTNP and MAVS-/-) was included in the model as an interaction term with viral load. Genes with a p-value of <0.05 were considered statistically significantly associated with viral load. Visualizations of the linear regression results were performed using the EnhancedVolcano and pheatmap packages in RStudio. Heatmap clustering for the top genes was done using Euclidean distance with average linkage.

### IFNγ release assay

PBMC from PWH cultured in RPMI 1640 supplemented with 10% FCS, penicillin (100 U/ml) and streptomycin (100 U/ml) alone or stimulated with either HIV-1 clade B gag peptide pool (2μg/ml; NIH AIDS reagent program) or CMV pp65 peptide pool (2μg/ml; NIH AIDS reagent program). In addition, MitoTEMPO (10uM; Sigma-Aldrich) or IL-12 (10ng/ml; Mitenyibiotec) were added to analyse the effect on IFNγ release. After 24 hours, culture supernatant was harvested and analysed for IFNγ by ELISA (Human IFN-gamma DuoSet ELISA; R&D Systems). The IFNg release assays were performed between August 31 and October 23, 2023.

### Intracellular cytokine staining

PBMC from PWH cultured in RPMI 1640 supplemented with 10% FCS, penicillin (100 U/ml), streptomycin (100 U/ml), anti-CD28 (2 μg/ml) and anti-CD29 (1 μg/ml) alone or stimulated with either HIV-1 clade B gag peptide pool (2μg/ml; NIH AIDS reagent program) or CMV pp65 peptide pool (2μg/ml; NIH AIDS reagent program). In addition, MitoTEMPO (10uM; Sigma-Aldrich) or IL-12 (10ng/ml; Mitenyibiotec) were added to analyse the effect on the number of cytokine producing cells and polyfunctionality. To all cultures, BD GolgiStop and BD GolgiPlug (BD Biosciences) was added. After 6 h incubation at 37 °C, cells were washed with PBS supplemented with 0.5% BSA and then stained extracellularly for 30 min at 4 °C in the dark with CD3 FITC (eBioscience), CD4 PerCP-Cy5.5 (BD Biosciences) and CD8 APC (Biolegend) followed by another wash step. For intracellular staining, cells were fixed and permeabilized using the BD cytofix/cytoperm kit (BD Biosciences) followed by staining for 30 min at 4 °C in the dark using the following fluorescently labelled antibodies: IFNγ PE, IL-2 BV421 and TNFα PE-Cy7 (Biolegend). PBMC were washed and fixed with CellFIX (BD Biosciences). Fluorescence was measured on the FACS Canto II (BD Biosciences) and marker expression levels were analysed using FlowJo Version 10.8.1 (TreeStar). Gating strategy is displayed in S7 Fig in S1 File. The intracellular cytokine staining were performed between August 31 and October 23, 2023.

### Statistical analysis

Statistical analysis on the participant characteristics were performed using a Mann-Whitney test in GraphPad Prism 8.3.0. P-values below 0.05 were considered statistically different. PCA on the virus-specific CD8 T cell transcription profiles was performed in IBM SPSS Statistics 26 using the logCPM values. Visualization of the PCA was performed using the RStudio ggplot2 package [47] with centroids depicting the median coordinates of the respective groups.

## Results

### Characteristics of study participants

HIV-infected individuals from 4 different groups were selected from the Amsterdam Cohort Studies on HIV infection and AIDS from whom stored peripheral blood mononuclear cell (PBMC) samples were available: progressors (n = 7), LTNP carrying the Human Leukocyte Antigen (HLA) B*57 allele (B*57 LTNP; n = 6), LTNP negative for HLA- B*57 (non-B*57 LTNP; n = 6) and individuals homozygous for the minor genotype of two linked SNPs (rs7262903 and rs7269320) in the MAVS gene that have previously been associated with virological control (MAVS-/-; n = 7) [39, 48]. In addition, CMV seropositive blood donors (BD) were included (n = 6). Characteristics of the HIV-infected participants are shown in Table 1. The age at seroconversion of the HIV-infected participants was comparable between groups. The number of months of AIDS-free untreated follow up was comparable between B*57-LTNPs and non-B*57 LTNP but B*57-LTNP had a significant longer AIDS-free follow up than progressors (p = 0.001) and MAVS-/- (p = 0.02). CD4 counts around the time-point of sampling were lower in progressors as compared to B*57 LTNP (p = 0.001), non-B*57 LTNP (p = 0.05) and individuals carrying the MAVS-/- albeit not significant (p = 0.199). The HIV RNA load in serum was significantly higher in progressors compared to B*57 LTNP (p = 0.015), but not non-B*57 LTNP (p = 0.66) and MAVS-/- (p = 0.16). Cell-associated viral loads in CD4 T cells were determined by qPCR or single genome amplification (SGA) using primers detecting pol sequences, and the cell-associated viral loads were significantly higher in progressors as compared to B*57 LTNP and MAVS -/- (p = 0.008 and 0.004 respectively) but not to non-B*57 LTNP (p = 0.23; Table 1).

**Table 1:** Participant characteristics.

	Progressor (n = 7)	B*57 LTNP (n = 6)	Non-B*57 LTNP (n = 6)	MAVS-/- (n = 7)
Age at HIV SC (yrs)	34.67 (25.42–46.33)	32.29 (28.54–43.08)	33.38 (27.42–34.92)	33.60 (21.38–35.85)
No. Participants developing AIDS	7/7	1/6	2/6	5/7
AIDS-free untreated follow up (mo.)	88.59 (77.21–114.39)	161 (122.07–278.39)*	156.1 (121.15–209.8)*	82.33 (61.87–271.05)
CD4 counts (cells/μl)**	300 (270–360)	635 (530–820)*	385 (310–510)	360 (170–510)
RNA load (10Log copies/ml)**	4.81 (3–5.47) ^$^	3 (1.78–4.23)^#^*	4.81 (3–5.09) ^$^	4.18 (3.35–4.85)
Cell-associated HIV load (copies/1x10^6^ cells)	18998 (4732–237819)	800 (20–12000)^^^*	11620 (600–31800)	2000 (400–7328)*

All values are median with minimum and maximum. Abbreviation: SC, Seroconversion; LTNP, long-term non-progressor.

**CD4 count and RNA load at the time of analysis.

^$^ 1 sample below the detection limit of the assay (1000 copies/ml)

^#^ 3 samples below the detection limit of the assay (1000 copies/ml)

^^^ 2 samples below the lower limit of quantification (<20 copies/1x10^6^ cells)

* Significantly different as compared to progressors (p < 0.05)

### Differential gene expression analysis of HIV-specific CD8 T cells from progressors and LTNP

**Fig 1:**
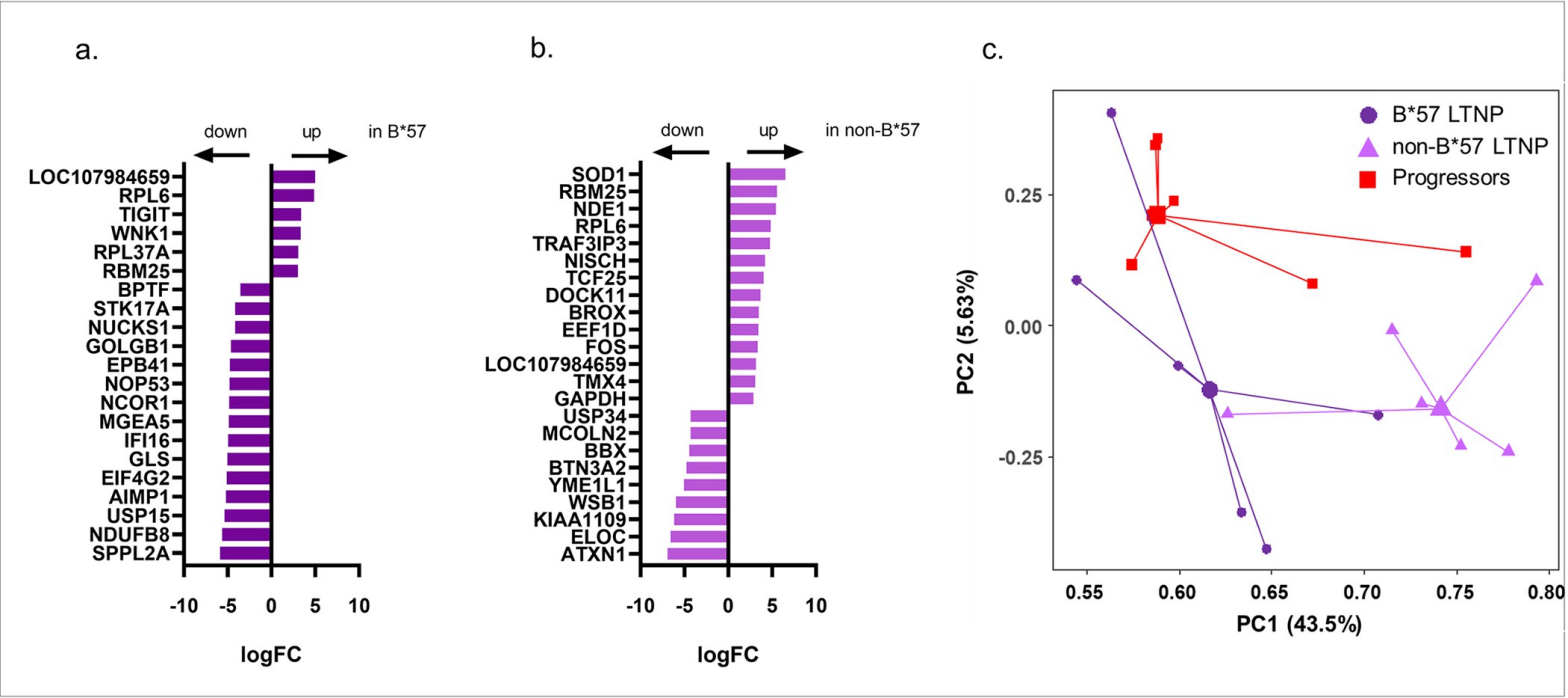
Differentially expressed genes in HIV-specific CD8 T cells and PCA plot. LogFC expression of the DEGs (logFC >1.5; P-value <0.05) in the HIV-specific CD8 T cells comparing (a) progressors versus B*57 long-term non-progressors (LTNP) and (b) progressors versus non-B*57 LTNP. Negative LogFC: upregulation of DEGs in progressors; Positive LogFC: upregulation of genes in B*57 LTNP, and non-B*57 LTNP as indicated. (c) PCA plot of the HIV-specific CD8 T cell transcription profiles from HIV progressors (red square), B*57 LTNP (purple circle) and non-B*57 LTNP (lilac triangle). Centroids are depicted by the larger symbols.

To determine differences in the transcriptional profile of HIV-specific CD8 T cells obtained during asymptomatic disease, from different groups of HIV infected individuals (progressors, HIV controller groups (B*57 LTNP, non-B*57 LTNP) and MAVS-/-) differential gene expression analysis was performed. When comparing the B*57 LTNP to the progressors 21 differentially expressed genes (DEGs) were found to be significantly different (log fold change (logFC) >1.5; P-value <0.05; Fig 1A; S2 Table in S1 Data) of which 6 DEG were upregulated in the B*57 LTNP. When comparing non-B*57 LTNP to progressors 23 DEGs were found of which 14 were upregulated in non-B*57 LTNP (Fig 1B; S3 Table in S1 Data). Three DEGs (RPL6, RBM25, LOC107984659) were upregulated in HIV-specific CD8 T cells from both B*57 LTNP and non-B*57 LTNP as compared to progressors.

Principal component analysis (PCA) of the transcription profiles of the HIV-specific CD8 T cells of B*57 LTNP, non-B*57 LTNP and progressors shows that the different groups of HIV infected individuals formed separate clusters (Fig 1C).

**Fig 2:**
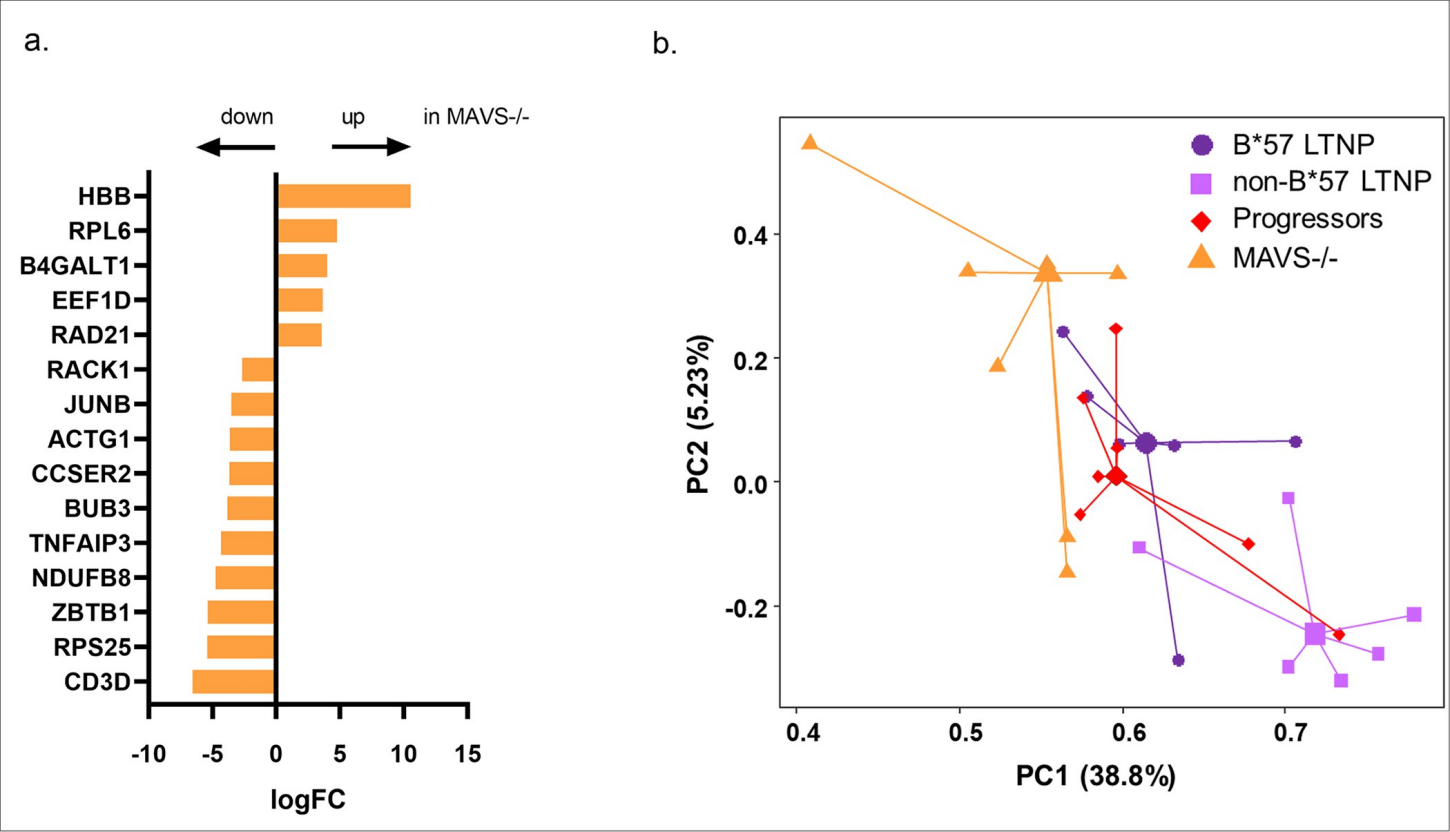
HIV-specific CD8 T cell transcription profile of MAVS -/- individuals differ from progressors, B*57 long-term non-progressors (LTNP) and non-B*57 LTNP. (a) LogFC expression of the DEGs comparing progressors versus MAVS -/- in the HIV-specific CD8 T cells (logFC >1.5; P-value <0.05). Negative LogFC: upregulation of DEGs in progressors; Positive LogFC: upregulation of genes in MAVS-/-. (b) PCA plot of the HIV-specific CD8 T cell transcription profiles from HIV progressors (red diamond), B*57 LTNP (purple circle), non-B*57 LTNP (lilac square) and MAVS -/- (orange triangle). Centroids are depicted by the larger symbols.

The differential gene expression analysis comparing HIV-specific CD8 T cells of progressors and MAVS -/- identified 15 DEGs (Fig 2A; S4 Table in S1 Data) of which the majority of the DEGs (10 DEGs) were downregulated in the MAVS -/- group. Among the genes that are differentially expressed were again ribosomal genes of which RPL6 was also upregulated in LTNP groups.

PCA including the MAVS -/- group shows these individuals form a separate cluster. This indicates that the HIV-specific CD8 T cells of the MAVS -/- carrying HIV infected individuals are not only distinct from the CD8 T cell transcription profile of the progressors but also from the B*57 LTNP and the non-B*57 LTNP (Fig 2B). In addition, this analysis shows that the gene expression profile of B*57 LTNP resembles that of the progressors, whereas the non-B*57 LTNP remain a distinct cluster.

To explore whether viral load could have influenced the gene expression differences between the groups, linear regression models were fitted to assess associations between gene expression and viral load. The multivariable analysis showed that 7 genes (ARPC2, RPS25, TPM3, RPL37, KMT2E, NDUFA13, DDX6) were statistically significantly associated with viral load (S1a Fig in S1 File). With the exception of RPS25 in the MAVS-/- participants no distinct clustering was observed for the genes among the different participant groups (S1b and S2 Figs in S1 File).

To identify whether the group specific DEGs are associated with cellular processes, Reactome pathway enrichment analysis was performed. When comparing progressors to B*57 LTNP, the identified DEGs were over-represented in 49 pathways (p < 0.05), including several pathways involving Nonsense Mediated Decay and protein translation (RPL37A, EIF4G2, RPL6; S5 Table in S1 Data). When comparing progressors to non-B*57 LTNP, only 22 pathways (p < 0.05) were identified, including pathways involving glucose metabolism and IL-12 signaling (SOD1, GAPDH; S6 Table in S1 Data). In both comparisons, pathways related to protein metabolism such as Eukaryotic Translation Elongation and Cellular responses to stress or stimuli were overrepresented (ELOC, FOS, RPL6, SOD1, NCOR1, RPL37A, EEF1D; S5 and S6 Tables in S1 Data).

Pathway enrichment analysis of DEGs in the progressor vs MAVS-/- comparison, identified 128 over-represented pathways (p < 0.05). Among the top pathways over-represented, again several of the DEGs play roles in pathways involved in Nonsense Mediated Decay and protein metabolism (Eukaryotic Translation Elongation; Peptide chain elongation; RPS25, RPL6, EEF1D; S7 Table in S1 Data). Moreover, pathways involved in immune system such as TNF and ALK Signaling (RACK1, TNFAIP3, JUNB; S7 Table in S1 Data) were identified.

**Fig 3:**
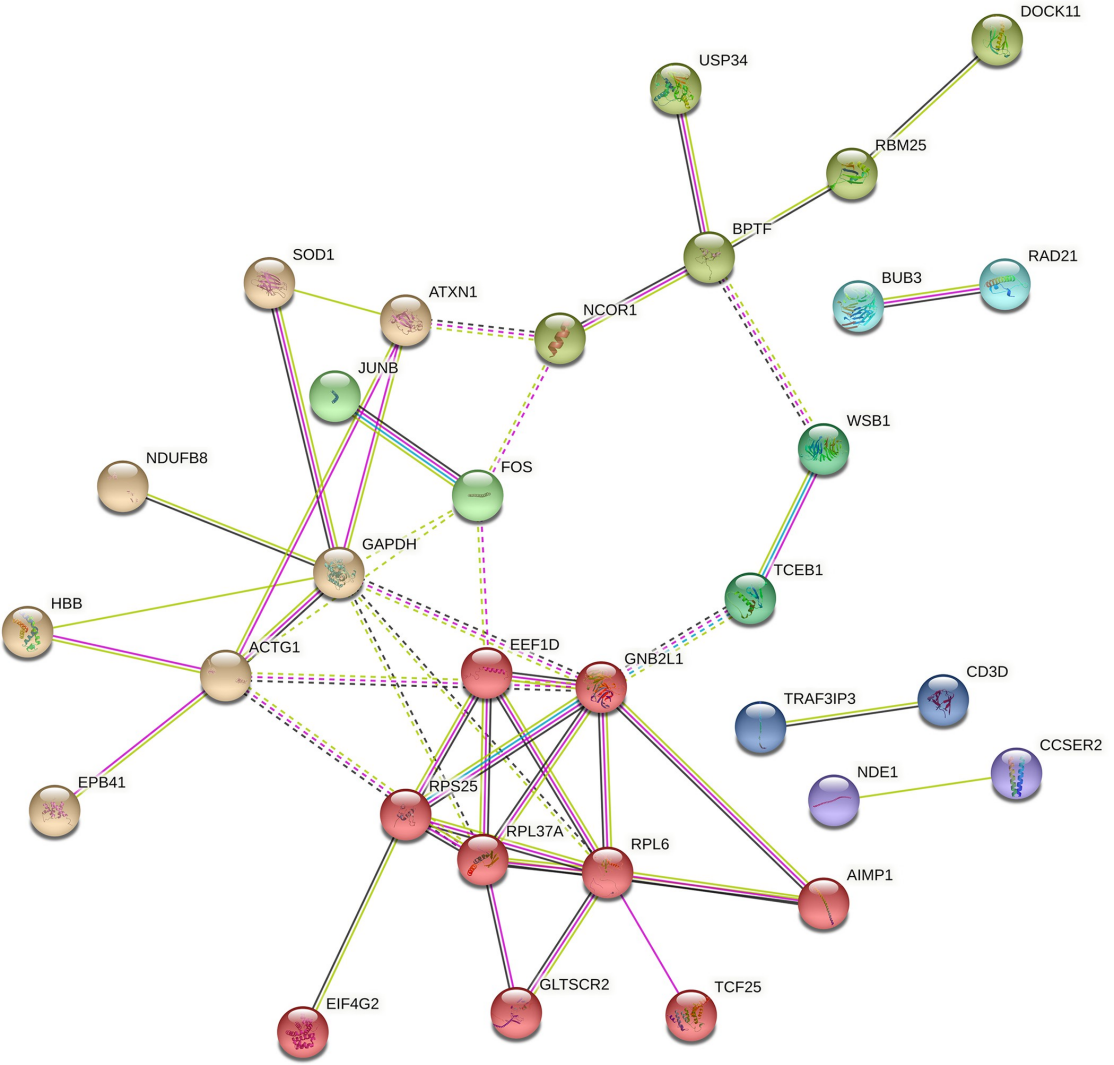
DEG network plot of the HIV-specific CD8 T cells. Network analysis of the DEGs in the HIV-specific CD8 T cells of HIV infected individuals reveals 8 different gene clusters.

Network analysis identified several distinct gene clusters with 31 nodes of known or predicted associations between the DEGs (Fig 3). The network revealed a relatively large cluster of genes involved in RNA and protein metabolism which became more pronounced when MAVS -/- were included in the analysis (S3 Fig in S1 File and Fig 3). In addition, a cluster containing genes involved in T cell receptor (TCR) signaling (CD3D, TRAF3IP3) and two clusters with genes associated with cell proliferation (BUB3, RAD21, NDE1, CCSER2), became more pronounced.

These data show that HIV-specific CD8 T cells from progressors and LTNP have a distinct gene expression profile and that these differences are mainly related to glucose metabolism, IL-12 signaling and the energy demanding RNA and protein metabolism.

### Differential gene expression analysis of CMV-specific CD8 T cells from HIV infected and uninfected individuals

To investigate whether HIV infection also affects CD8 T cells specific for other antigens, the gene expression profile of CMV-specific CD8 T cells was analyzed in the same HIV-infected individuals (progressors (n = 7), B*57 LTNP (n = 3), non-B*57 LTNP (n = 6), MAVS -/- (n = 7)). For comparison an additional group of CMV-positive BD was included (n = 6).

**Fig 4:**
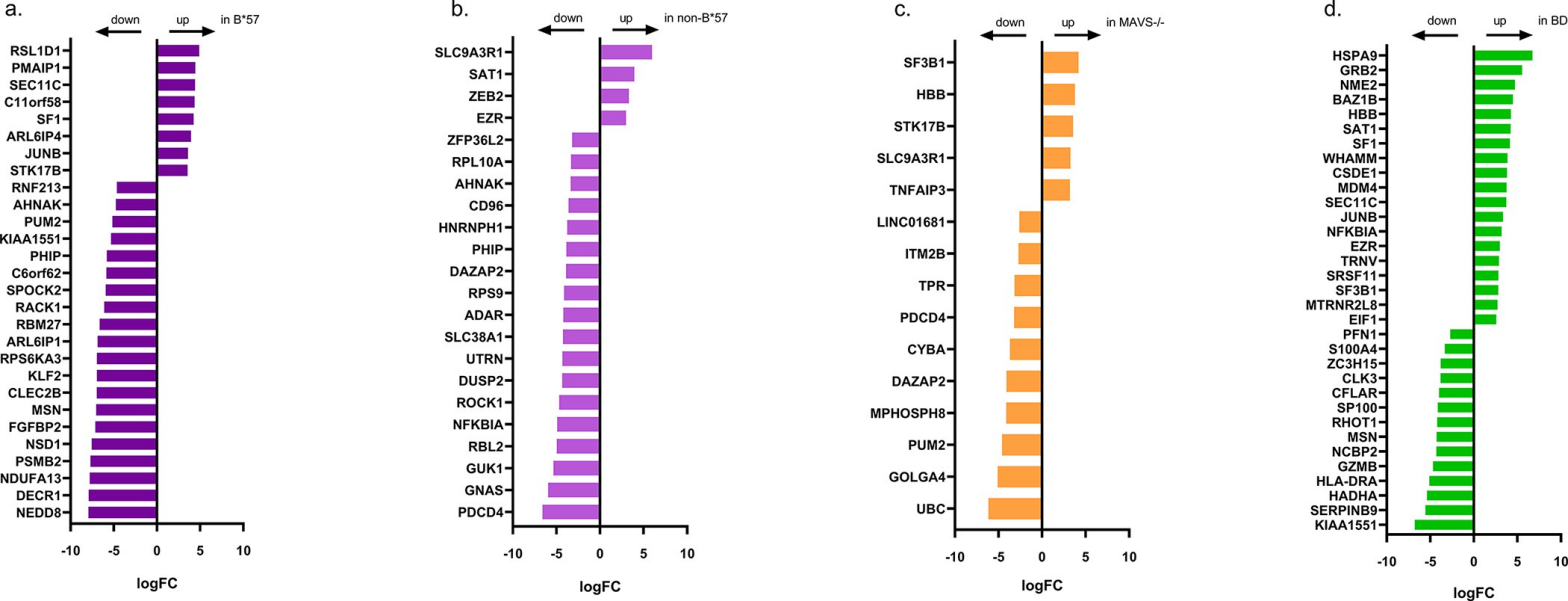
Differentially expressed genes CMV-specific CD8 T cells. LogFC of the DEGs (logFC >1.5; P-value <0.05) in the CMV-specific CD8 T cells when comparing progressors versus: (a) B*57 long-term non-progressors (LTNP), (b) non-B*57 LTNP, (c) MAVS-/- and (D) BD. Negative LogFC: upregulation of DEGs in progressors; Positive LogFC: upregulation of genes in B*57 LTNP, non-B*57 LTNP, MAVS-/- and BD as indicated.

Differential gene expression analysis identified 28 and 22 DEGs (logFC >1.5; P-value <0.05; S8 and S9 Tables in S1 Data) when comparing the CMV-specific CD8 T cells of B*57 or non-B*57 LTNP to progressors respectively (Fig 4A and 4B), of which the majority was downregulated in B*57 LTNP (20 DEGs) or non-B*57 (18 DEGs) LTNP. In CMV-specific T cells obtained from MAVS -/-, 15 DEGs were observed when compared to progressors (S10 Table in S1 Data), of which 10 were downregulated in MAVS -/- (Fig 4C). In CMV-specific CD8 T cells from BD, a total of 33 DEGs were found when compared to progressors, of which 14 were downregulated in BD (Fig 4D; S11 Table in S1 Data).

**Fig 5:**
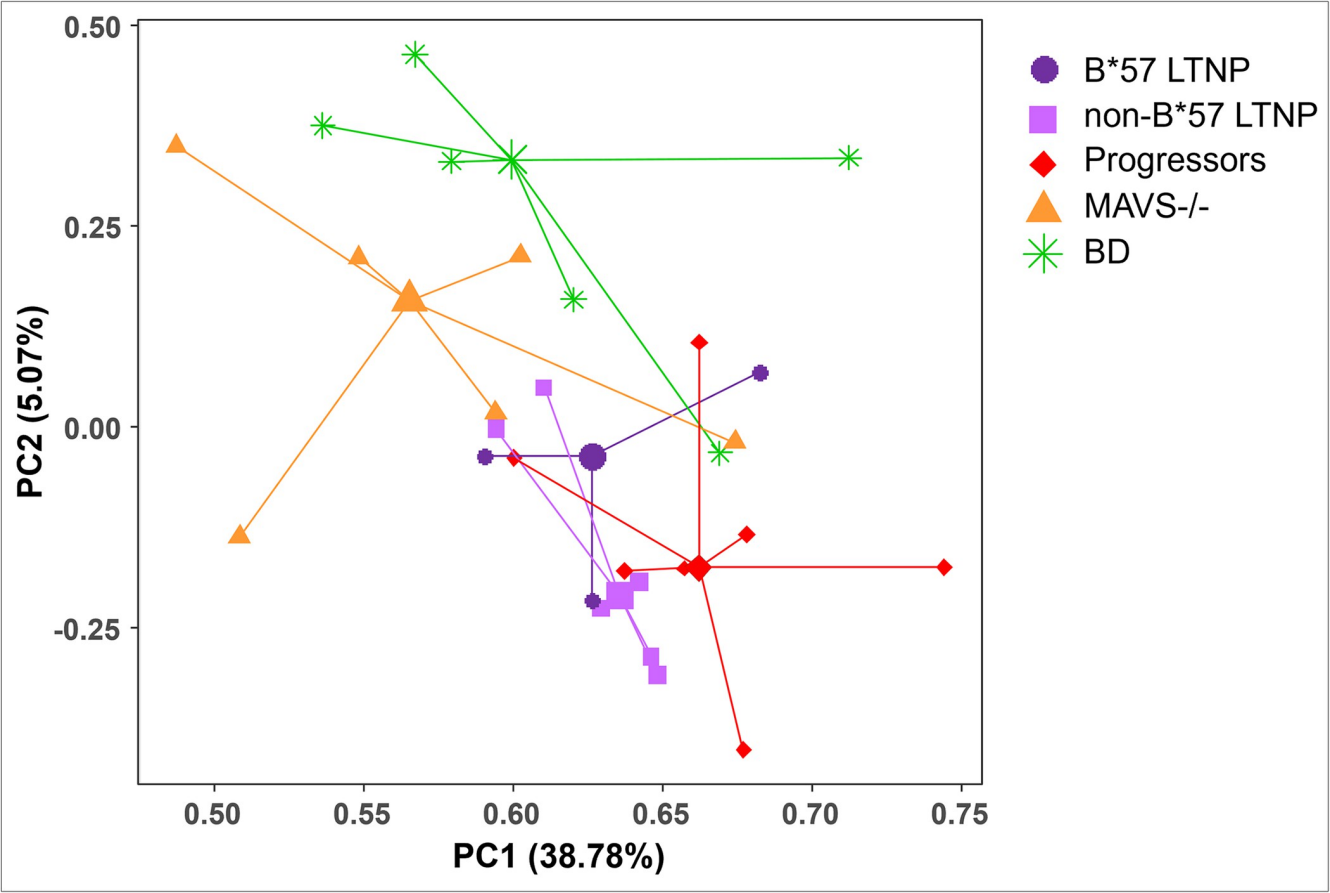
CMV-specific CD8 T-cell transcription profile of BD differ from MAVS -/-, progressors, B*57 long-term non-progressors (LTNP) and non-B*57 LTNP. PCA plot of the CMV-specific CD8 T cell transcription profiles from BD (green asterisk), HIV progressors (red diamond), B*57 LTNP (purple circle), non-B*57 LTNP (lilac square) and MAVS -/- (orange triangle). Centroids are depicted by the larger symbols.

PCA showed that the transcriptional profiles of CMV-specific CD8 T cells from BD and MAVS -/- were distinct and clustered separately from the other groups of HIV infected individuals (progressors, B*57 and non-B*57 LTNP; Fig 5).

Pathway analysis of DEGs in CMV specific CD8 T cells in B*57 LTNP versus progressors, revealed over-representation of 35 pathways (p< 0.05). Among these were pathways involved in ALK signaling (RNF213, MSN, JUNB; S12 Table in S1 Data) and cytokine (IL-12 and TGF-B) signaling (RPS6KA3, PSMB2, MSN, NEDD8, JUNB; S12 Table in S1 Data). In contrast, the enriched pathways in non-B*57 LTNP versus progressors (73 pathways; p< 0.05; S13 Table in S1 Data) were related to protein/RNA metabolism such as Nonsense-mediate decay (NMD) (RPS9, HNRNPH1, ADAR, RPL10A). In the pathway analysis of the DEGs comparing MAVS -/- to progressors (153 pathways; p< 0.05; S14 Table in S1 Data) the TNF and IL-12 -signaling (UBC, TNFAIP3, PDCD4) and antiviral mechanisms (TPR, UBC) pathways were enriched. In BD versus progressors, 78 over-represented pathways were identified (p< 0.05; S15 Table in S1 Data), involving ALK and cytokine signaling (MSN, GRB2, HSPA9, NFKBIA, SP100, MSN, HLA-DRA, GZMB, GRB2, JUNB) and RNA metabolism (NCBP2, SF3B1, SRSF11, SF1).

**Fig 6:**
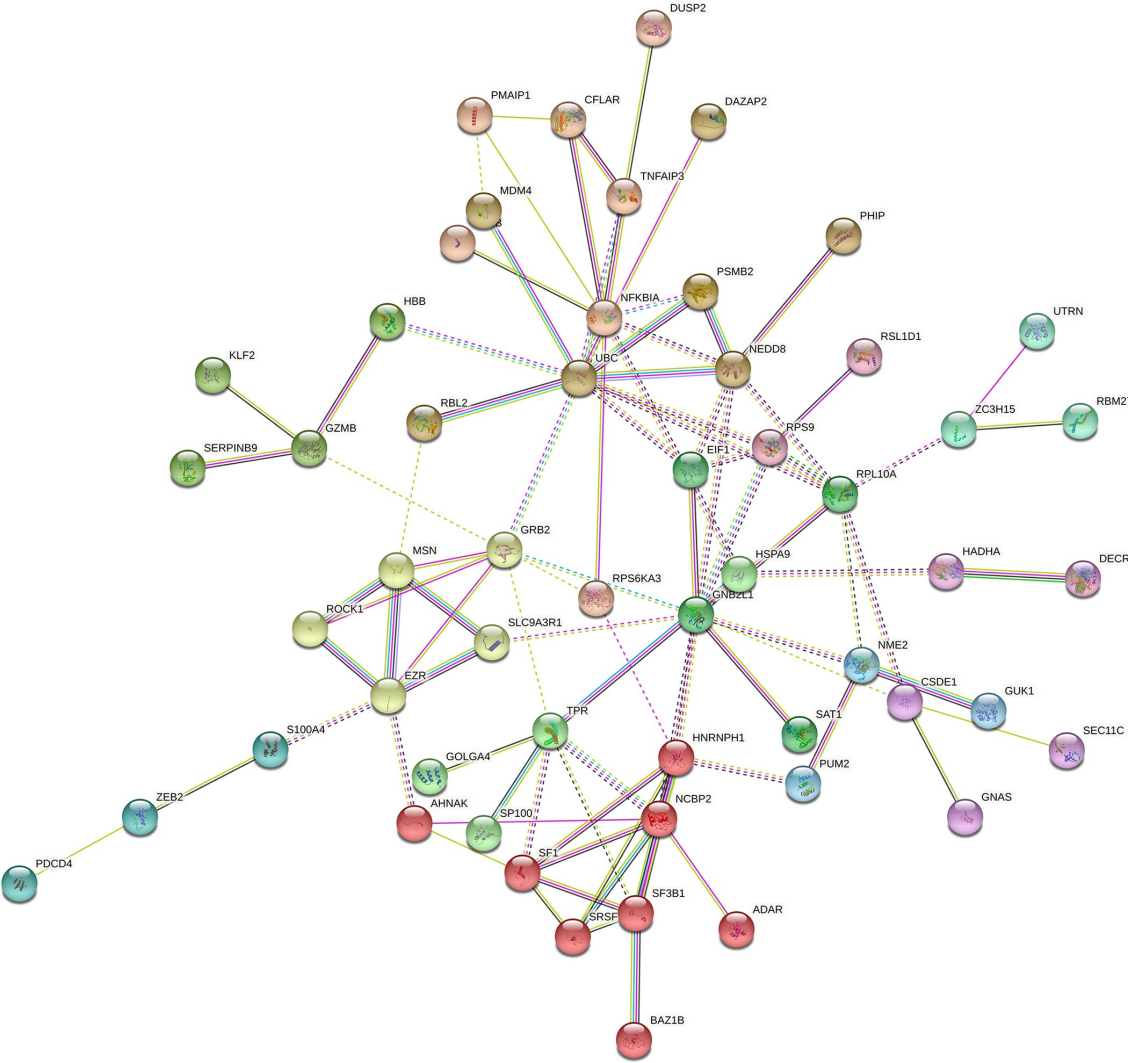
DEG network plot of CMV-specific CD8 T cells. Network analysis of the DEGs found in the CMV-specific CD8 T cells of HIV infected individuals and BD reveal 13 different gene clusters.

Network analysis of the DEGs found in the CMV-specific CD8 T cells from HIV infected individuals reveals a relatively large clusters of genes involved in signal transduction and RNA metabolism (S4 Fig in S1 File). Conversely, a cluster with T cell functionality/effector genes (KLF2, GZMB, SERPINB9) became more pronounced when BD were included in the analysis (Fig 6 and S4 Fig in S1 File).

These data show that differential gene expression of CMV-specific CD8 T cells observed in progressors is mainly related to upregulation of genes involved in cytokine signaling and the energy demanding RNA and protein metabolism.

### Distinct transcriptional profiles of HIV- and CMV-specific CD8 T cells from HIV infected individuals

**Fig 7:**
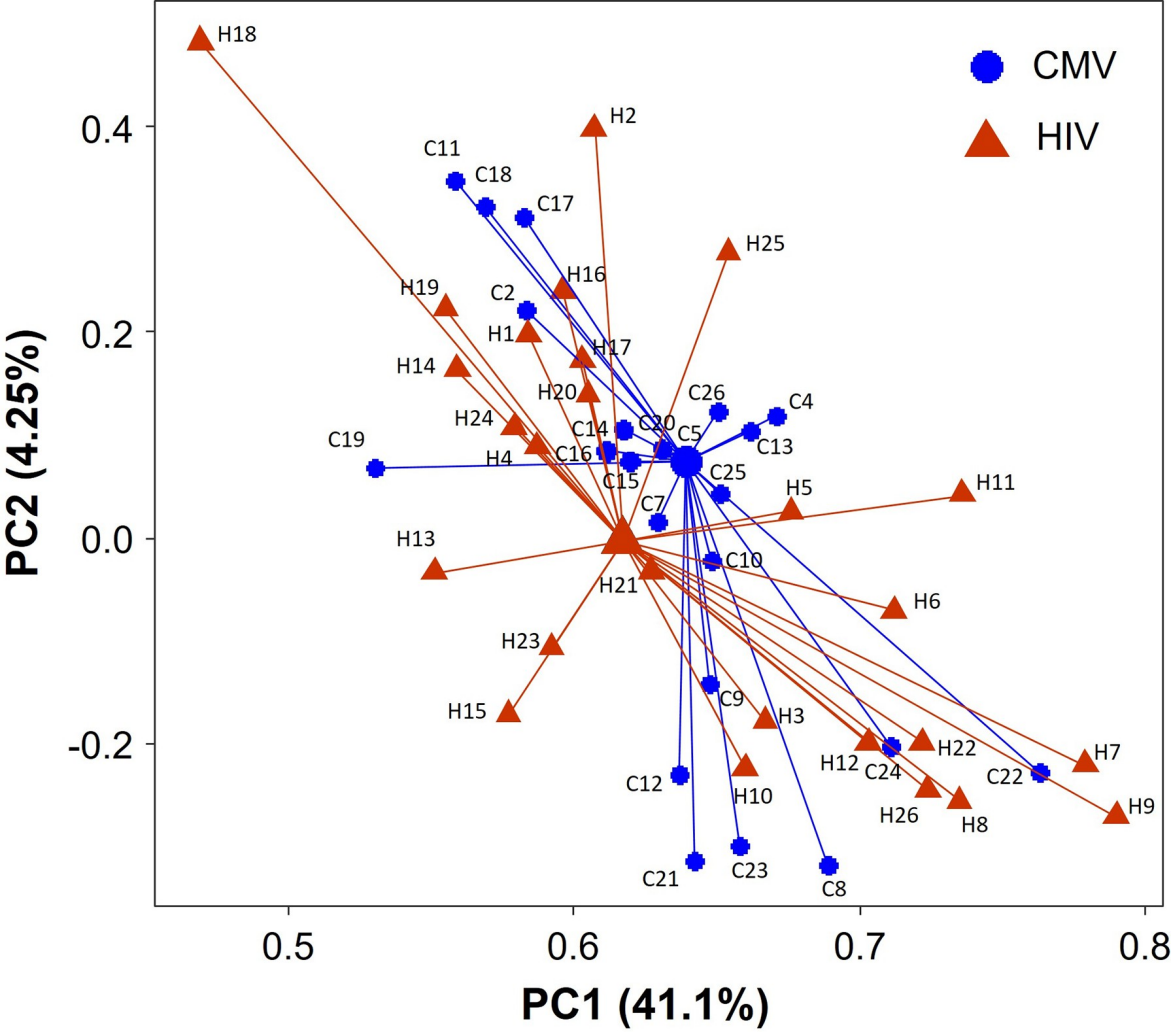
HIV- and CMV-specific CD8 T cells of HIV infected individuals have a different transcriptional profile. PCA plot of the transcription profile of HIV-specific CD8 T cells (brown triangle) and CMV-specific CD8 T cell (blue circle) from HIV infected individuals. Centroids are depicted by the larger symbols. Each individual is indicated by a number, whereas a letter is given for HIV (H) or CMV (C) specific CD8 cells.

Pathway analysis suggest that the mRNA expression profile differed between CMV- and HIV-specific CD8 T cells from HIV infected individuals. Indeed, PCA demonstrated that HIV- and CMV-specific CD8 T cells isolated from the same HIV infected individual clustered separately (Fig 7). A comparative gene expression analysis is shown in S16-19 Tables in S1 Data, however most observed differences in gene expression were lost upon multiple comparison correction. Moreover, pathway analysis demonstrated that distinct differential gene expression of HIV- and CMV-specific CD8 T cells between progressors and LTNP are related to similar processes including RNA and protein metabolism and cytokine signaling (S5-S7 and S12-S14 Tables in S1 Data).

### Targeting mitochondrial dysfunction improves IFNγ production upon HIV- and CMV-peptide stimulation

Transcriptional profiling of HIV- and CMV-specific CD8 T cells from progressors and LTNP demonstrated differential expression of genes related to the energy demanding RNA and protein metabolism as well as cytokine signaling. Recently it has been shown that the energy demands of T cells upon antigen stimulation, require a metabolic switch towards glycolysis and subsequent mitochondrial respiration to maintain their functionality [49, 50]. Moreover, antigen specific T cell responses also rely on mitochondria for ROS production and signaling [35].

To determine whether mitochondrial dysfunction may be the underlying course of the aberrant gene expression profile in antigen specific CD8 T cells and their dysfunctionality in chronic HIV infection, we analyzed whether mitochondrial targeting would improve T cell functionality. Previously, the anti-oxidant piperidine-nitroxide MitoTempo and IL-12 have been demonstrated to increase mitochondrial polarization and restore the effector function of T cells [51, 52].

**Fig 8:**
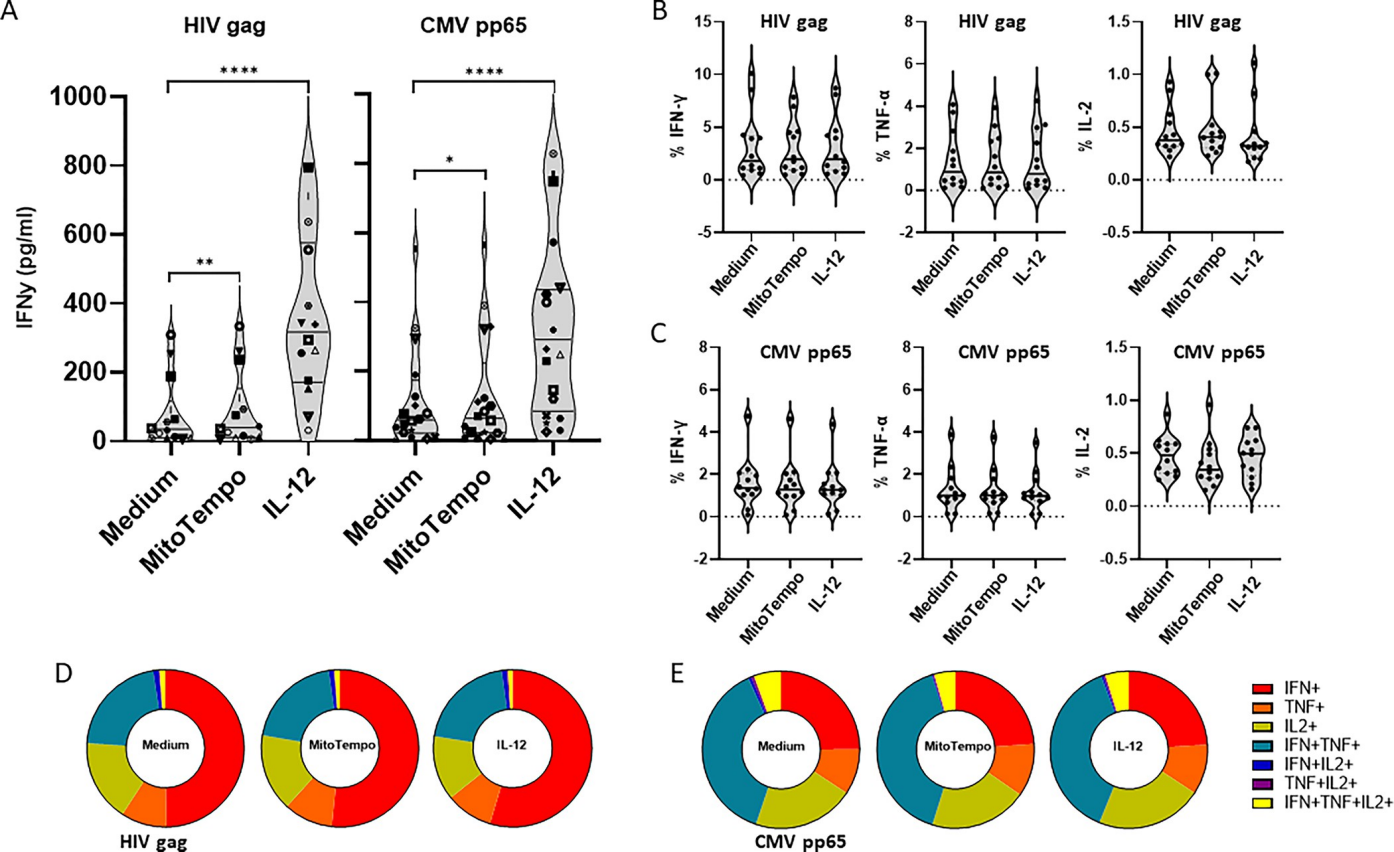
Targeting mitochondrial dysfunction improves cytokine release by HIV- and CMV-specific cells from HIV infected individuals. **A**. IFNγ release upon HIV gag (left panel; n = 14) and CMV pp65 (right panel; n = 20) peptide pool stimulation in the presence of MitoTempo or IL-12. Symbols indicate data points from the same individual. Intracellular cytokine staining for IFNγ, IL-2 and TNFα (B and C) and polyfunctionality (D and E) upon HIV gag (B and D) and CMV pp65 (C and E) peptide pool stimulation in the presence of MitoTempo or IL-12 (n = 12). P-value < 0.05 (*), 0.01 (**), 0.001 (***), 0.0001(****).

To this extent, PBMC from PWH (characteristics described in S5 Fig in S1 File) were stimulated with HIV gag and CMV pp65 peptide pools in the presence or absence of MitoTempo and IL-12. T cell functionality was determined by IFNγ release and intracellular cytokine staining. MitoTempo and IL-12 significantly increased IFNγ release upon stimulation with HIV gag as well as CMV pp65 (Fig 8A). In contrast to previous observations in chronic hepatitis B [51, 52], in PWH the percentage of IFNγ, TNFα and IL-2 producing CD8 T cells did not increase in the presence of MitoTempo and IL-12 (Fig 8B and 8C). Moreover, MitoTempo and IL-12 also did not increase the amount of IFNγ, TNFα and IL-2 produced per CD8 T cell (S6A and S6B Fig in S1 File). We also observed that MitoTempo and IL-12 treatment had no effect on the polyfunctionality of the HIV gag or CMV pp65 specific CD8 T cells (Fig 8D and 8E). These data show that targeting of mitochondria by MitoTempo and IL-12, improved virus specific CD8 T cell functionality by increasing the capacity of these cells to release of cytokines upon antigen recognition. Similar observations were made for CD4 T cells stimulated with HIV gag or CMV pp65 peptide pools (S7 Fig in S1 File).

## Discussion

Using RNA-sequencing we have determined the transcriptional profiles of HIV- and CMV-specific CD8 T cells isolated from PBMC samples from HIV-infected individuals. We observed that the transcriptional profiles of HIV-specific CD8 T cells obtained from progressors, B*57 LTNP, and non-B*57 LTNP were unique for each group and formed three separate clusters in the PCA.

The gene expression profile of HIV-specific CD8 T cells from progressors was associated with decreased functionality with regards to RNA and protein metabolism, in agreement with previous observations [53], and may be indicative of a more exhausted phenotype. Exhaustion of T cells is associated with up-regulation of inhibitory markers, such as PD-1, LAG-3, TIM-3 and CTLA-4 and has been linked to T cell dysfunction and disease progression as well as viral rebound after treatment interruption and size of the viral reservoir [24, 27, 54–56]. Moreover, gene set enrichment analysis in a previous study demonstrated that the gene expression profile of HIV-specific CD8 T cells from progressors was similar to the exhaustion profile found in LCMV-specific CD8 T cells in a mouse model for chronic LCMV infection [53]. Another study showed opposing transcriptional profiles comparing the HIV-specific central memory CD8 T cells of HIV controllers and ART treated non-controllers, with upregulation of genes related to activation and exhaustion in non-controllers [57]. Here, we did not observe increased expression of genes related to exhaustion of HIV-specific T cells in progressors, nor in CMV-specific CD8 T cells when comparing progressors to LTNP or healthy donors. This discrepancy might at least in part be caused by differences in the participant selection, as we analysed the transcriptional profile of HIV-specific CD8 T cells before the onset of disease progression and when the overall T cell dysfunction was less severe. Our findings are in accordance with a previous study that also did not find hallmarks of canonical exhaustion in longitudinal transcriptional profiling of functionally impaired HIV-specific CD8 T cells prior to loss of control [58]. Moreover, in this study they found that expression of genes related to cell cycle regulation were altered. In our network analysis, a cluster of genes associated with mitosis (BUB3, RAD21) was identified. In contrast to Collins et al. [58], who found upregulation of an anti-proliferative factor in individuals with loss of control, we found increased expression of mitotic checkpoint protein BUB3 in progressors as compared to MAVS-/-. The increased expression of BUB3 suggests increased proliferation of HIV-specific CD8 T cells in progressors, which could be indicative of recent antigen exposure and high CD8 T cell turnover and contribute to immune activation, exhaustion and immune dysfunction [59]. Moreover, the altered bioenergetic metabolism of exhausted T cells [33] may also affect the transcriptional profile especially of genes related to RNA and protein metabolism. The distinct group specific gene-expression profile was not driven by the viral load. We found that a unique set of genes was associated with viral load, which had no overlap with the group DEGs, with the exception of RPS25 which was also found to be differentially expressed in the MAVS-/- participants.

In our analysis, 2 groups of LTNP (B*57 and non-B*57 LTNP) were included who are able to control their HIV infection for an extended period. Based on the transcriptional profile of the HIV-specific CD8 T cells the LTNP groups clustered separately in the PCA, but the transcriptional profile of B*57 LTNP was more comparable to that of progressors. This indicates that HIV infection in B*57 LTNP is not solely controlled by a strong CD8 T cell response. Indeed, it has previously been demonstrated that viral escape from HLA-B*57 restricted CD8 T cells is associated with viral attenuation resulting in lower plasma and cellular viral load which substantially contributes to viral control and slow disease progression [16, 60–62]. The transcription profile of HIV-specific CD8 T cells from non-B*57 LTNP indicated increased functionality as compared to progressors and B*57 LTNP. However, the distinct transcription profile of non-B*57 LTNP in combination with the higher viral RNA load as compared to B*57 LTNP suggest that HIV is controlled without a severe attenuating effect on viral replication. Indeed, different genetic polymorphisms in genes related to the immune system may also affect the functionality of CD8 T cell response [39, 63–65] and may at least in part explain the increased CD8 T cell functionality in these non-B*57 LTNP. Recently, it has been suggested that viral adaptation to the immune response can also modulate the HIV-specific CD8 T cell transcription profile and functionality [66], and may therefore also play a role in disease progression. They observed that cross-reactive CD8 T cells exhibited a transcriptional profile with increased effector functions after recognition of wild type epitope as compared to recognition of an adapted epitope containing immune escape mutations [66]. These data indicate a role for the autologous virus in maintenance or loss of CD8 T cell functionality during chronic HIV infection.

HIV-specific CD8 T cells from individuals homozygous for the minor genotype of two linked SNPs (rs7262903 and rs7269320) in the MAVS gene (MAVS -/-) which has previously been associated with a delayed viral load incline over the course of infection [48, 67], showed a distinct transcription profile when compared to the other HIV-infected groups, and formed a separate cluster in the PCA. Again, the DEGs were associated with protein metabolism and immunological pathways and are indicative of increased function of HIV-specific CD8 T cells in MAVS -/-. The MAVS-/- genotype has previously been associated to insensitivity of the MAVS protein to the PLK1-dependent suppression by HIV resulting by a robust type I IFN response upon DDX3 triggering [67]. The induction of a robust innate response may also affect the adaptive immune response resulting in increased functionality of HIV-specific CD8 T cells. Moreover, the MAVS -/- genotype has previously been shown to decrease HIV replication in PBMC most likely due to the induction of an antiviral response in dendritic cells and monocyte/macrophages [48].

Transcription profile analysis of CMV- and HIV-specific CD8 T cells from the same HIV-infected individuals, revealed almost no overlap of DEGs. When comparing the transcriptional profiles of CMV-specific CD8 T cells between the different groups of HIV-infected individuals, we observed that most of the identified DEGs were upregulated in progressors as compared to B*57 LTNP, non-B*57 LTNP or MAVS -/-. Pathway analysis showed that DEGs were enriched in signal transduction, protein/RNA metabolism and immune system related pathways. Moreover, CMV-specific CD8 T cells of progressors show upregulation of genes associated with effector functions (GZMB, SERPINB9) as compared to healthy BD. Upregulation of these genes could be a consequence of recent or frequent reactivation of CMV in HIV progressors as previously reported [68–70]. PCA showed that the transcription profile of CMV-specific CD8 T cells from the BD and MAVS -/- clustered separately from the other groups of HIV-infected individuals (progressors, B*57 LTNP and non-B*57 LTNP). This indicates an increased effector function of CMV-specific T cells in the progressors as well as LTNP possibly reflecting activation of the CMV-specific immune response in these groups. In HIV-infected individuals with the MAVS -/- genotype, CMV-specific CD8 T cells showed a different transcription profile more comparable to healthy BD. This may indicate that the CMV-specific immune response is less frequently activated in the HIV-infected individuals with the MAVS -/- genotype than in progressors and the LTNP. Indeed, it has been suggested that double stranded RNA produced during viral transcription can activate MAVS-dependent signaling cascades via RIG-I [71, 72]. Although the effect of the MAVS -/- genotype on MAVS-dependent signaling by CMV RNA transcripts is not known, the high similarity of the CMV-specific CD8 T cell transcription profile with that of healthy donors suggests better control of CMV.

Although the transcriptional profile differed between CMV- and HIV-specific CD8 T cells within the groups of PWH, pathway analysis showed that the transcriptional alteration occurred in several common pathways including cytokine/IL-12 signaling and the energy demanding protein/RNA metabolism. CD8 T cell functionality upon antigen recognition, is dependent on metabolic reprogramming to meet the high energy demands of cellular proliferation, differentiation, and effector functions like cytokine production [49, 50]. Mitochondria play an important role in the bioenergy metabolism as well as ROS-dependent signaling essential for the antigen-specific activation of T cells [35]. Mitochondrial dysfunction may be the underlying course of the aberrant gene expression profile in antigen specific CD8 T cells and their dysfunctionality in chronic HIV infection, as has been shown previously in chronic HIV [73] and HBV infection [51, 52]. Indeed, improving mitochondrial function by the anti-oxidant piperidine-nitroxide MitoTempo and IL-12 increased IFNγ release upon stimulation with HIV gag as well as CMV pp65, but did not affect the percentage of IFNγ, TNFα and IL-2 producing CD8 T cells as was observed in chronic hepatitis B [51, 52].

Our study has several limitations: The sample size is limited due to the use of historical cryopreserved PBMC samples from treatment naïve participants of the Amsterdam Cohort Studies. These samples are rare and have limited cell numbers for analysis. Some inter-individual variation in the transcriptional signatures may have occurred due to differences in the amount of cells available for analysis, however for majority of the samples the same amount was used so we expect this to be limited. Due to the limited availability of the samples, it was also not possible to perform additional functional analysis. For the isolation of CMV and HIV-specific CD8 T cells, we used well-known immune dominant epitopes and we were able to detect CD8 T cells recognizing these epitopes in all study participants. It cannot be excluded that our study may not provide a complete picture of the transcriptional profile of all virus-specific CD8 T cells. However, it should be noted that we were able to show distinct clustering of the different groups of HIV-infected individuals based on the transcription profiles. In this study, the transcriptional profiles were determined of HIV-specific CD8 T cells isolated from peripheral blood. However, lymphoid tissue has been shown to be a main site of HIV replication [74–76] and recently it has been shown that tissue resident HIV-specific CD8 T cells in this tissue are the main effector cell to control HIV replication [77, 78]. The transcription profile of circulating CD8 T cells is distinct from their tissue resident counterparts [77]. Although differences in the functionality of circulating CD8 T cells have been linked to prolonged control of HIV replication [12–22], it should be noted that our analysis in circulating CD8 T cells may not be representative of the functionality of tissue resident HIV-specific CD8 T cells. Due to the cross-sectional nature of our study, changes in the transcriptional profile of HIV- and CMV-specific CD8 T cells during the course of infection could not be analysed. In addition, it should be stated that after correcting for multiple comparisons, many of the DEG’s found did not remain significant thus caution has to be taken not to overinterpret the transcriptional profiles.

In summary, we were able to distinguish the transcriptional profiles of HIV and CMV-specific CD8 T cells from HIV progressors, B*57 LTNP, non-B*57 LTNP and individuals with the MAVS -/- genotype. The transcriptional profile of HIV-specific CD8 T cells obtained during the asymptomatic phase of infection, from progressors was indicative of impaired functionality related to RNA and protein metabolism, whereas the transcriptional profile of CMV-specific CD8 T cells was associated increased effector functions. Our data shows that changes in cytokine signaling and the energy demanding RNA and protein metabolism may be indicative of mitochondrial dysfunction and drive dysfunctionality of virus-specific T cells. Targeting the mitochondria to improve their function, indeed improved IFNγ release upon antigen stimulation. Therefore, novel treatment strategies aiming to enhance the cellular metabolism and improve mitochondrial function may improve virus = specific CD8 T cell responses and aid a controlling immune response in chronic infection.

## Supporting information

S1 Data S1-S19 Tables can be found in the excel file. (XLSX)

S1 File S1-S9 Figs can be found in the pdf file. (PDF)
