# Supplementary material for: Energy demanding RNA and protein metabolism drive dysfunctionality of HIV-specific T cell changes during chronic HIV infection
Source: PLoS One. 2024 Oct 2;19(10):e0298472. doi: 10.1371/journal.pone.0298472 (PMC11446443; doi:10.1371/journal.pone.0298472)

Supporting information

Energy demanding RNA and protein metabolism drive dysfunctionality of HIV-specific T cell changes during chronic HIV infection

Lisa van Pul[1,2], Melissa Stunnenberg[1,2], Stefanie Kroeze[1,2,3#], Karel A. van Dort[1,2], Brigitte D. M. Boeser-Nunnink[1,2], Agnes M. Harskamp[1,2], Teunis B. H. Geijtenbeek[1,2], Neeltje A. Kootstra[1,2*]

[1] Amsterdam UMC location University of Amsterdam, Department of Experimental Immunology, Meibergdreef 9, Amsterdam, The Netherlands

[2] Amsterdam Institute for Infection and Immunity, Amsterdam, The Netherlands

*** Correspondence:**

N.A. Kootstra

n.a.kootstra@amsterdamumc.nl

Content:

Supplementary figures

Supplementary Figure S1. Multivariable linear regression analysis to explore genes associated with viral load. Volcano plot of the multi variable linear regression results (a). Heatmap of genes that were associated with viral load, counts are standardized within rows. Clustering was done using Euclidean distance with average linkage.

Supplementary Figure S2. Correlation plots of genes associated with viral load. Each dot represent the $\log_{10}$ CPM gene count and $\log_{10}$ viral load.

Supplementary Figure S3. DEG network plot of the HIV-specific CD8 T cells of progressors and long-term non-progressors (LTNPs). Network analysis of the DEGs in the HIV-specific CD8 T cells of progressors and LTNPs (B*57 & non-B*57) reveals 5 different clusters.

Supplementary Figure S4. DEG network plot of the CMV-specific CD8 T cells of progressors, long-term non-progressors (LTNPs) and MAVS-/-. Network analysis of the DEGs in the HIV-specific CD8 T cells of progressors, LTNPs (B*57 & non-B*57) and MAVS-/- reveals 8 different clusters.

Supplementary Figure S5. **Participant characteristics analysed for intracellular cytokine production and IFN-γ release.**

Supplementary Figure S6. **Mean fluorescence intensity analysis of IFN-γ, TNF-α and IL-2 of HIV (A) and CMV (B) specific CD8 T cells cultured in the presence of medium, Mito Tempo or IL-12.**

Supplementary Figure S7. Intracellular cytokine staining for IFN- γ, IL-2 and TNFα shown as % cytokine producing CD4 T cells (A), mean fluorescence intensity analysis (B) and polyfunctionality (C) upon HIV gag and CMV pp65 peptide pool stimulation in the presence of MitoTempo or IL-12.

Supplementary Figure S8. **Flow cytometry gating strategy for the (identification and) sorting of HIV and CMV-specific CD8 T cells.**

Supplementary Figure S9. **Flow cytometry gating strategy for intracellular cytokine staining of HIV and CMV specific CD8 T cells.**

Supplementary Figure S1. Multivariable linear regression analysis to explore genes associated with viral load. Volcano plot of the multi variable linear regression results (a). Heatmap of genes that were associated with viral load, counts are standardized within rows. Clustering was done using Euclidean distance with average linkage.

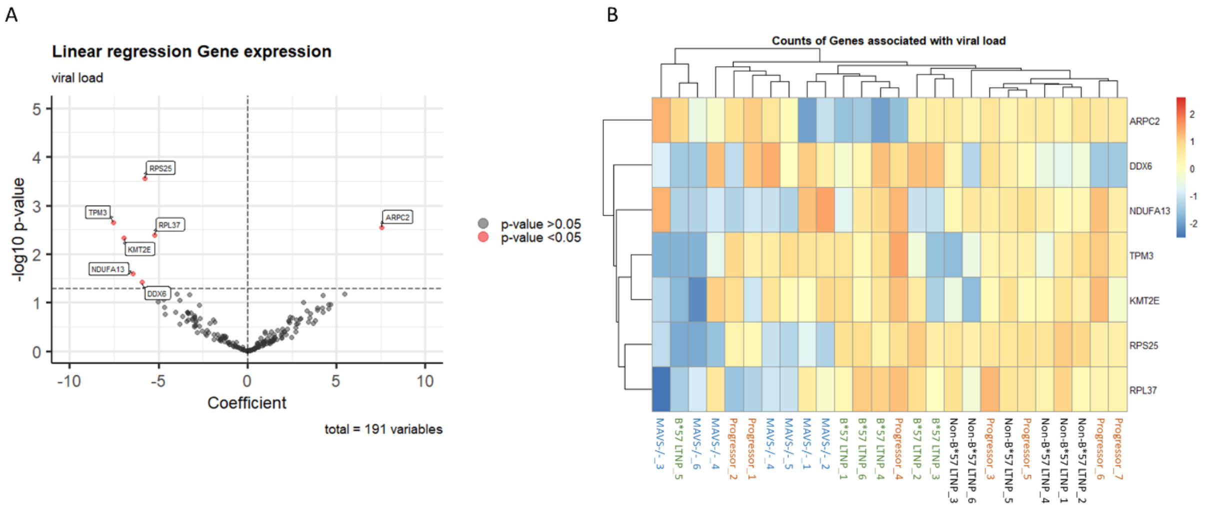

Supplementary Figure S2. Correlation plots of genes associated with viral load. Each dot represent the $\log_{10}$ CPM gene count and $\log_{10}$ viral load.

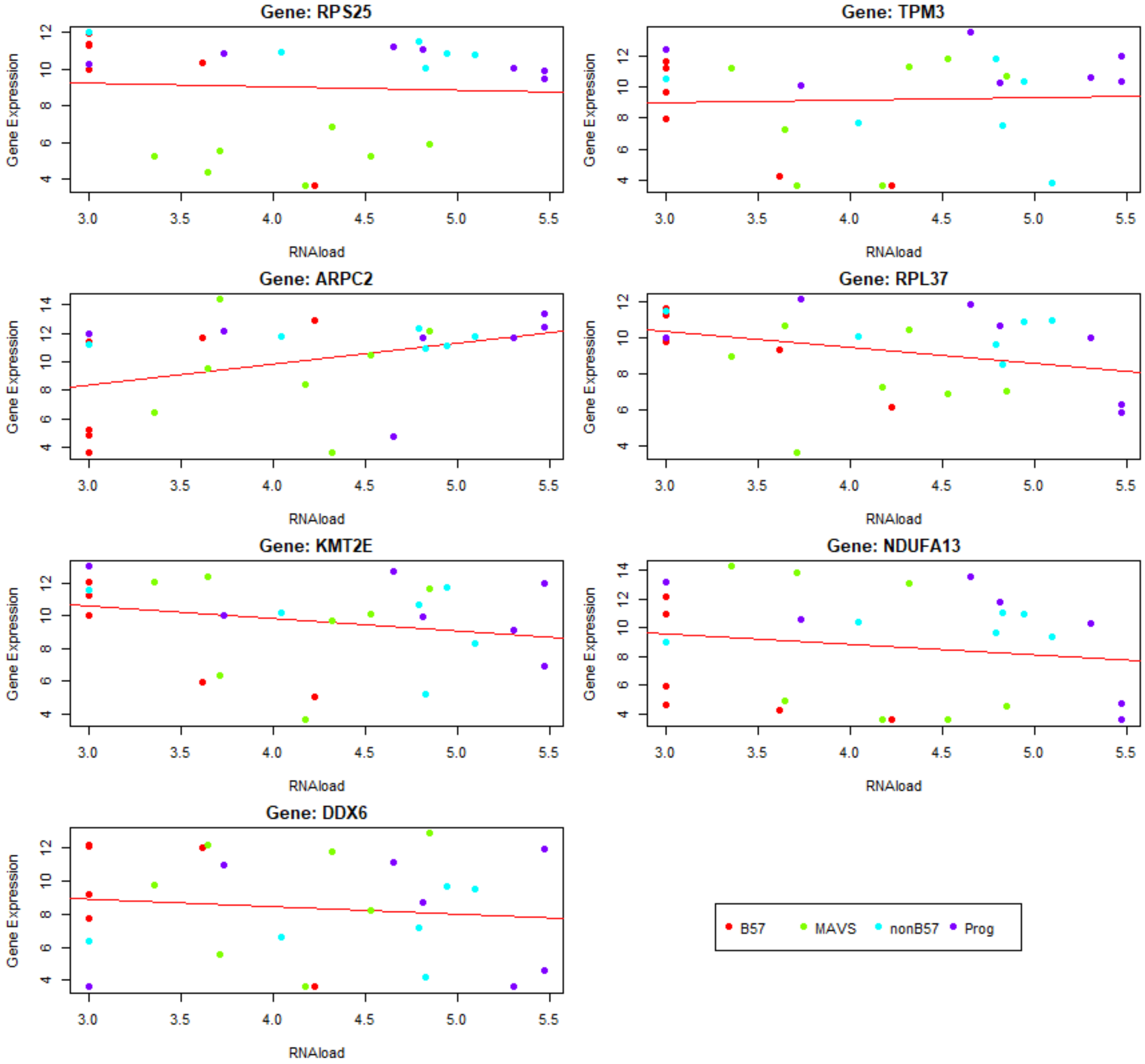

Supplementary Figure S3. DEG network plot of the HIV-specific CD8 T cells of progressors and long-term non-progressors (LTNPs). Network analysis of the DEGs in the HIV-specific CD8 T cells of progressors and LTNPs (B*57 & non-B*57) reveals 5 different clusters.

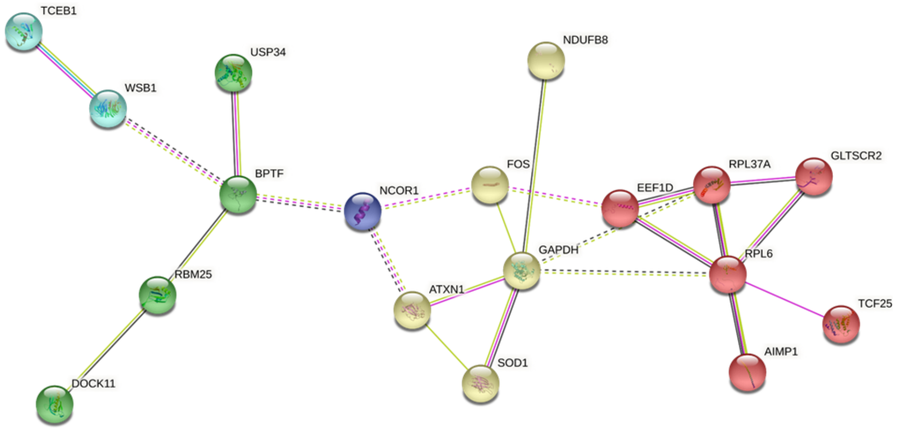

Supplementary Figure S4. DEG network plot of the CMV-specific CD8 T cells of progressors, long-term non-progressors (LTNPs) and MAVS-/-. Network analysis of the DEGs in the HIV-specific CD8 T cells of progressors, LTNPs (B*57 & non-B*57) and MAVS-/- reveals 8 different clusters.

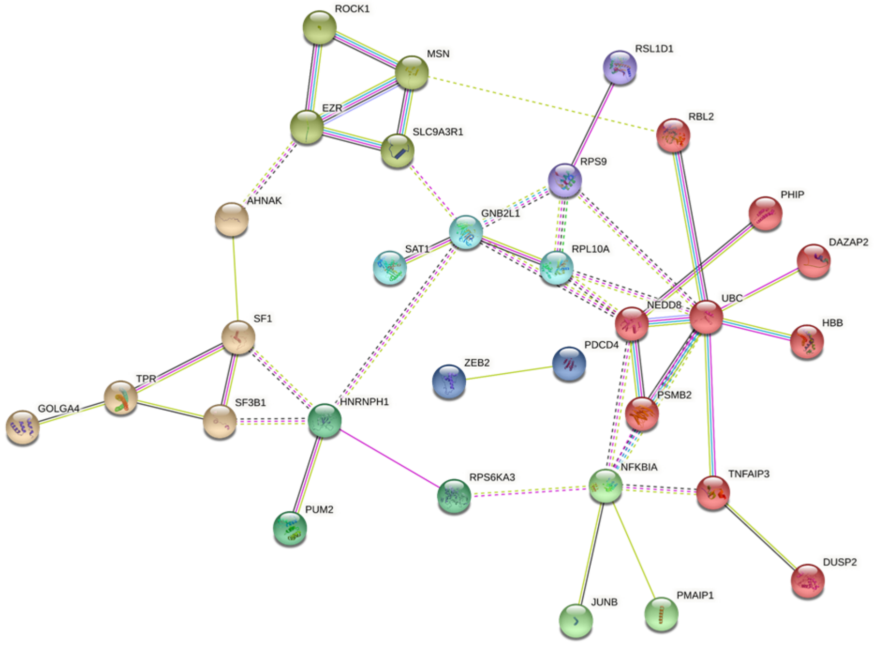

Supplementary Figure S5. Participant characteristics analysed for IFN-γ release assay and intracellular cytokine staining.

	**Median (IQR)**
No. Participants	20[*]
Age at HIV SC (yrs)	32.4 (29.5-37.1)
Time after HIV SC (yrs) [**]	3.9 (3.5-5.2)
CD4 counts (cells/μl)[**]	455 (343-618)
RNA load (^{10}Log copies/ml)[**]	34500 (3475-63750)

[*]6 individuals showed no HIV gag response; For 2 participants cell numbers were insufficient for the intracellular cytokine production.

[**]at the time of analysis.

Supplementary Figure S6. **Mean fluorescence intensity analysis of IFN-γ, TNF-α and IL-2 of HIV (A) and CMV (B) specific CD8 T cells cultured in the presence of medium, Mito Tempo or IL-12.**

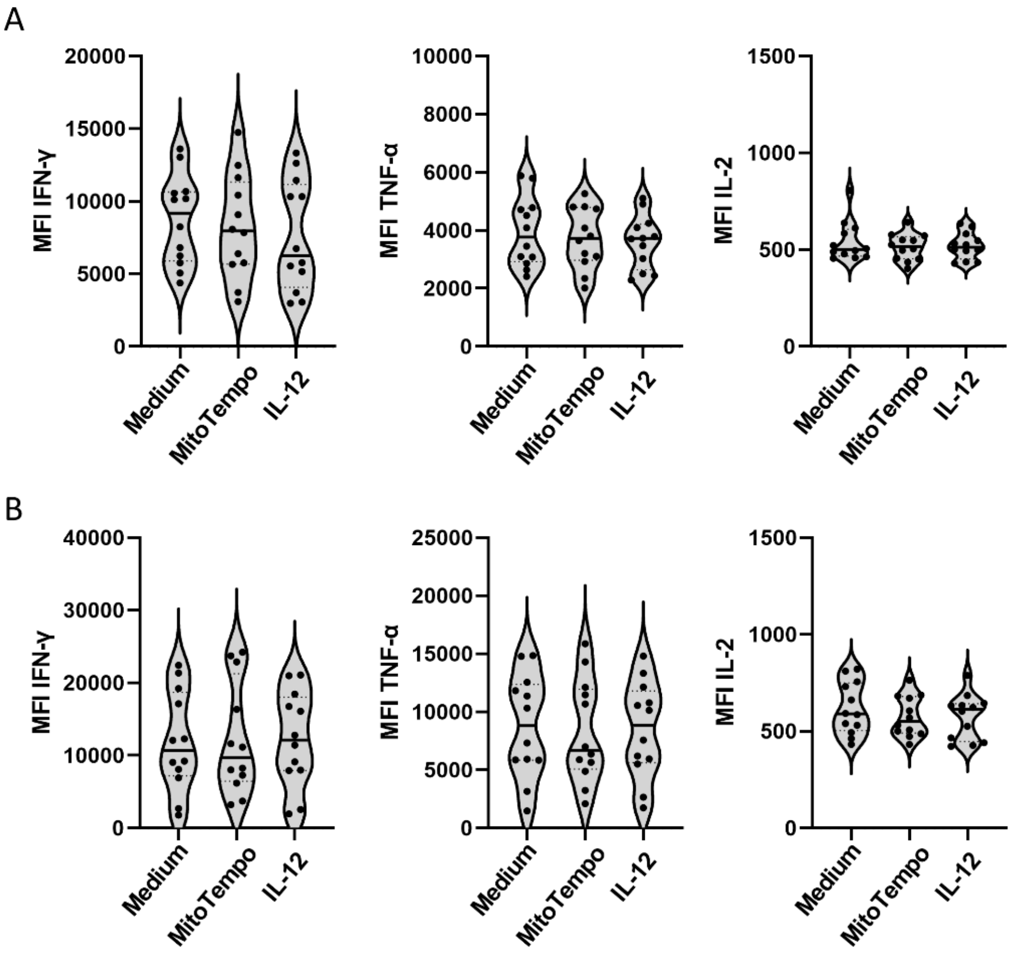

Supplementary Figure S7. Intracellular cytokine staining for IFN- γ, IL-2 and TNFα shown as % cytokine producing CD4 T cells (A), mean fluorescence intensity analysis (B) and polyfunctionality (C) upon HIV gag and CMV pp65 peptide pool stimulation in the presence of MitoTempo or IL-12.

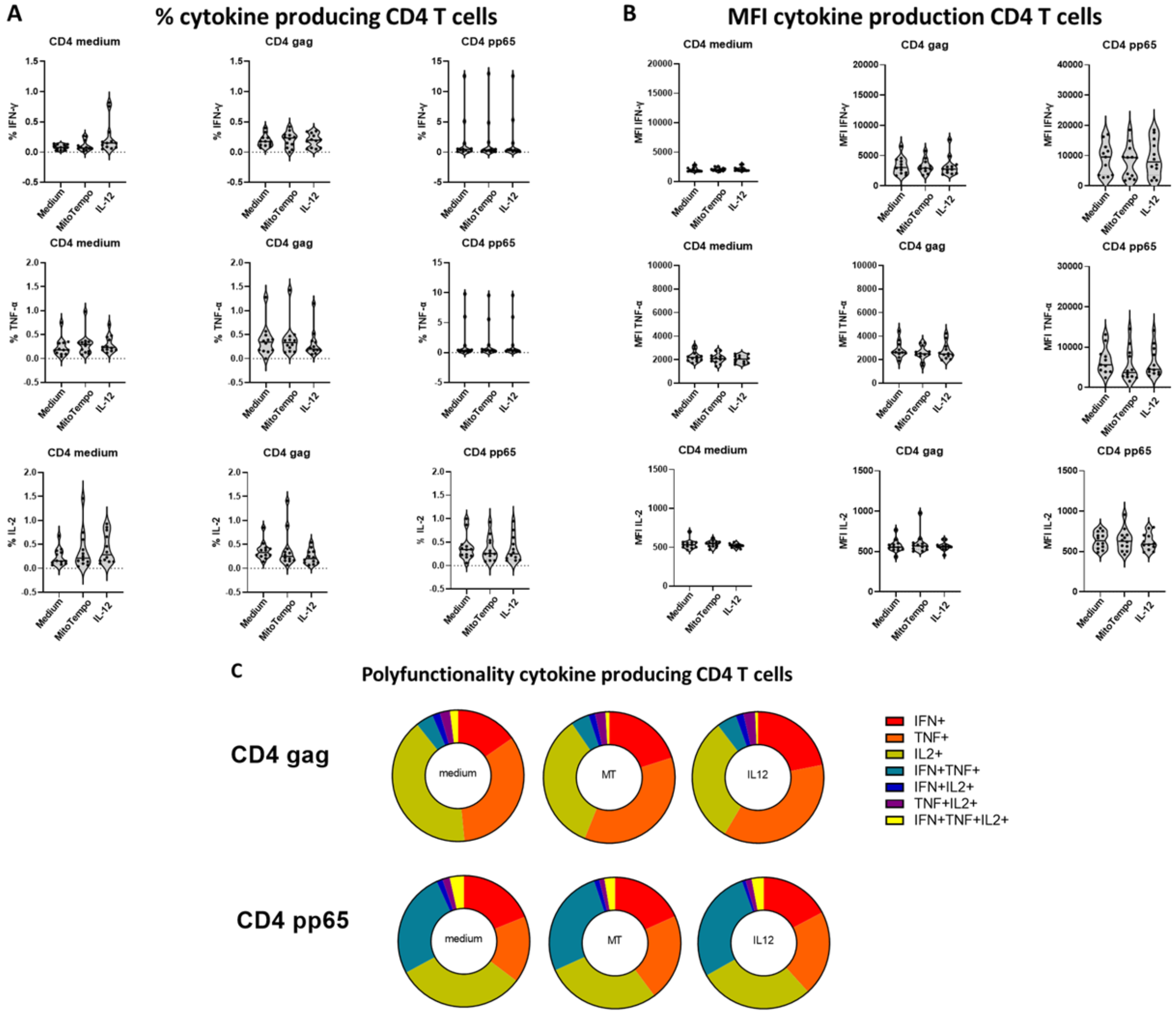

Supplementary Figure S8. **Flow cytometry gating strategy for the (identification and) sorting of HIV and CMV specific CD8 T cells.**

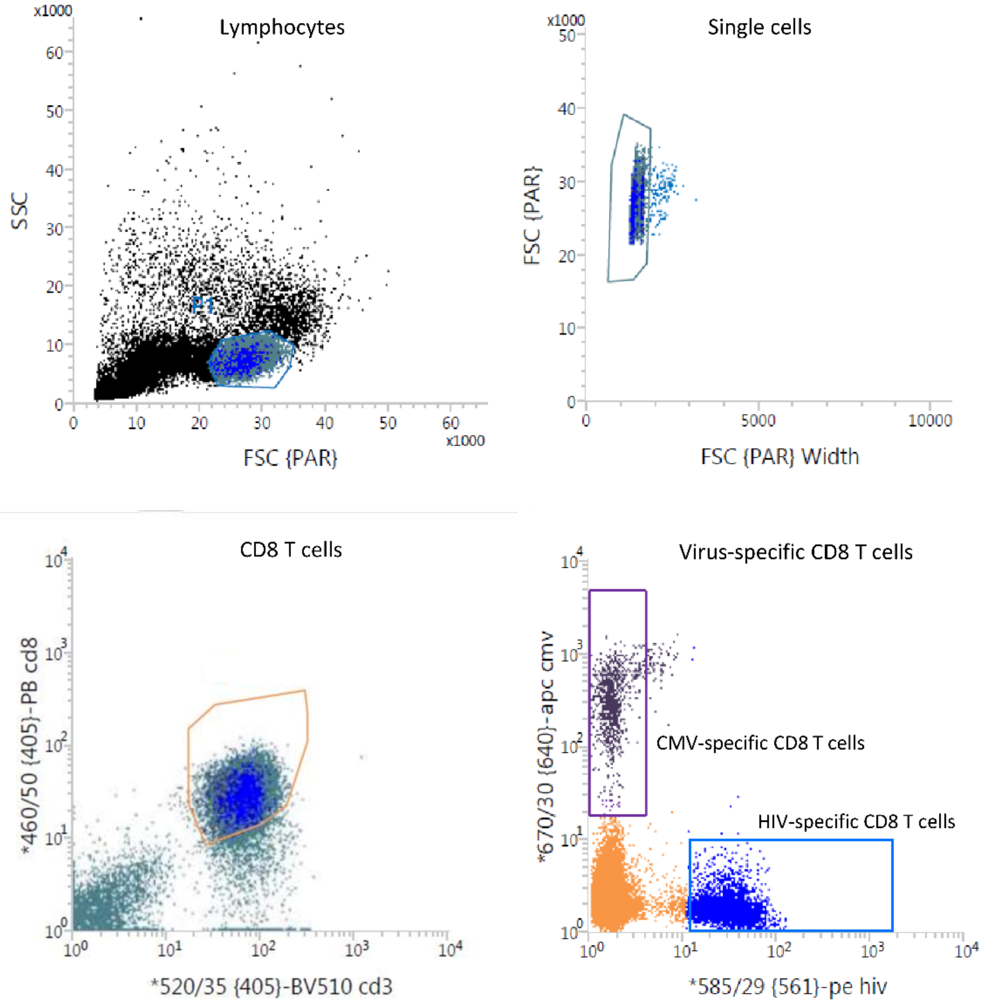

Supplementary Figure S9. **Flow cytometry gating strategy for intracellular cytokine staining of HIV and CMV specific CD8 T cells.**

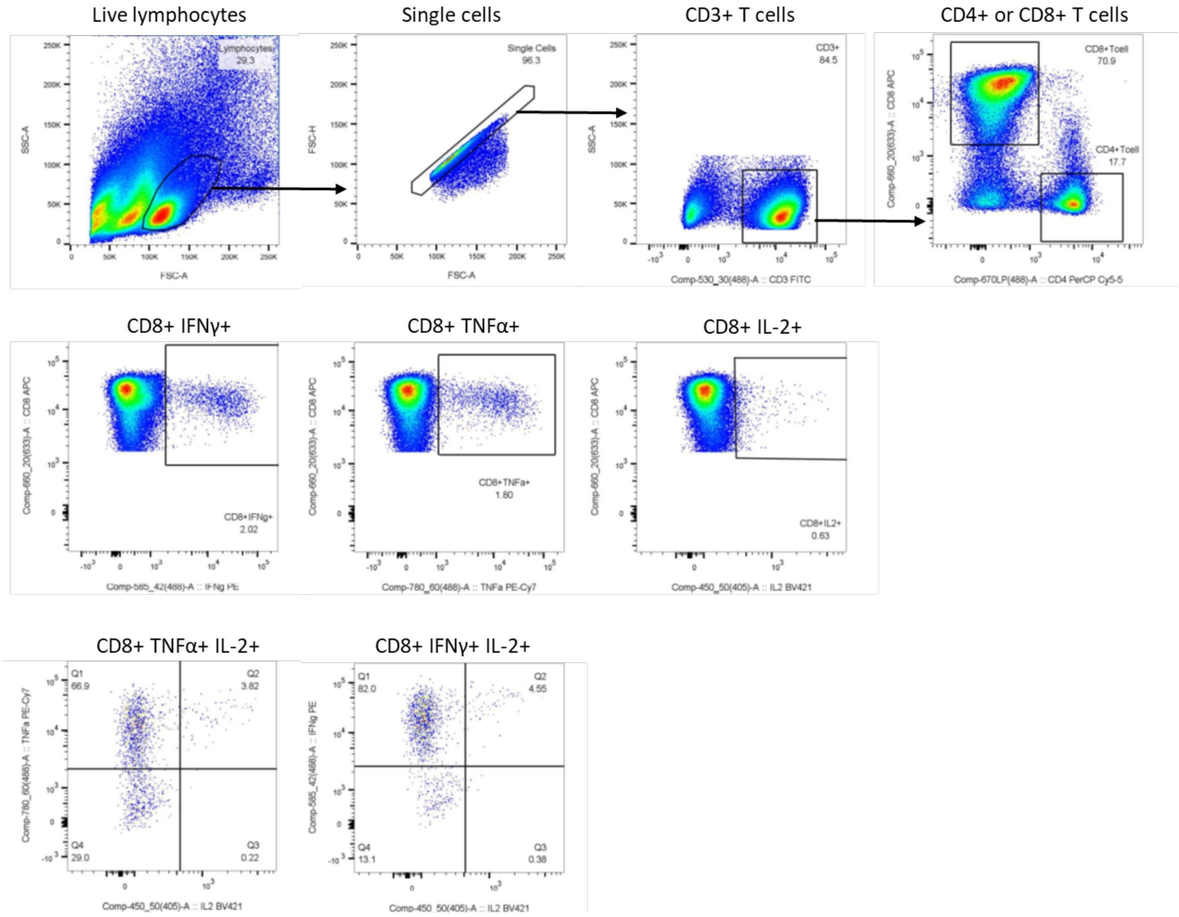

Supplement: S1 File — (PDF) [file pone.0298472.s002.pdf]
